# Minimally Invasive Posterior Spinal Nonfusion Surgery in Patients With Adolescent Idiopathic Scoliosis Using a Bipolar One-Way Self-Expanding Rod System: Protocol for a Single-Center Clinical Cohort Study

**DOI:** 10.2196/47222

**Published:** 2023-12-25

**Authors:** Anne Mareille Post, Hanneke I Berends, Barend J van Royen

**Affiliations:** 1 Department of Orthopedic Surgery and Sports Medicine University Medical Center Amsterdam Amsterdam Netherlands; 2 Amsterdam Movement Sciences Amsterdam Netherlands; 3 Emma Children’s Hospital University Medical Center Amsterdam Amsterdam Netherlands

**Keywords:** adolescent idiopathic scoliosis, minimally invasive, nonfusion, posterior spinal surgery, protocol, one-way self-expanding rod, scoliosis, idiopathic scoliosis, spinal fusion, spinal deformity surgery, nonfusion techniques, surgery, intraoperative, surgical technique, operation, musculoskeletal, orthopedic

## Abstract

**Background:**

The current surgical treatment for patients diagnosed with progressive and severe adolescent idiopathic scoliosis (AIS) consists of the correction of the spinal curvature, followed by posterior spinal fusion (PSF). However, research has uncovered short- and long-term complications of posterior spinal fusion in patients with AIS. Minimally invasive growing rod techniques have successfully been used to treat patients with early-onset scoliosis and neuromuscular scoliosis. It may be questioned if minimally invasive posterior spinal nonfusion (PSnF) surgery with bipolar instrumentation can be used for the treatment of AIS.

**Objective:**

This study will be performed to monitor the efficacy and safety of PSnF surgery by using a commercially available Conformité Européenne-certified spinal implant consisting of bilateral bipolar one-way self-expanding rods (OWSER) for the treatment of patients diagnosed with AIS.

**Methods:**

In 14 selected patients with AIS with Lenke 1-6 curves, minimally invasive PSnF surgery with the OWSER system is performed after the failure of conservative treatment (curve progression of >5° within 1 year). The patients are over 7 years of age, with a major Cobb angle of ≥30°, sufficient flexibility, and a Risser stage of ≤2. Patients will be followed over time, according to the standard medical care. Efficacy will be measured using radiological and patient satisfaction assessments and safety will be determined by the amount of perioperative complications.

**Results:**

Patient inclusion started on November 17, 2021 and we hope to finalize patient inclusion by the beginning of 2025. The first results will be expected by the beginning of 2024.

**Conclusions:**

Minimally invasive PSnF in patients with AIS is presented as a less invasive surgical technique that prevents the progression of the scoliotic curve and that allows minor posture correction of coronal imbalance. This will be the first study to examine whether the PSnF bipolar OWSER instrumentation will be the next generation of surgical instrumentation in AIS.

**Trial Registration:**

ClinicalTrials.gov NCT04441411; https://clinicaltrials.gov/study/NCT04441411

**International Registered Report Identifier (IRRID):**

DERR1-10.2196/47222

## Introduction

Adolescent idiopathic scoliosis (AIS) is a 3D deformity of the spine that is clearly associated with the adolescent growth spurt [1]. Up to 10% of patients diagnosed with AIS require some form of treatment, and almost 0.3% will eventually undergo corrective spinal surgery [2]. When the scoliotic curve exceeds 40° to 45°, surgical correction followed by posterior spinal fusion (PSF) is regarded as the standard surgical treatment for AIS [3,4].

Both short- and long-term complications of PSF have been described in relation to AIS, such as spinal imbalance, implant failure, infection and wound complications, degenerative disk disease (16%), postoperative pain, distal adding-on, and proximal junctional kyphosis [5-10]. These complications all result in additional treatment or possibly a reduced quality of life. Therefore, there is a need for a type of minimally invasive spinal surgery that allows natural growth, enables spontaneous spinal balance correction and prevents full spinal fusion to improve the outcomes of patients with AIS.

Minimally invasive nonfusion spinal surgery in AIS is not new. Anterior vertebral body tethering and a concave distraction technique (ApiFix) have been used as nonfusion techniques in patients with AIS, although with mixed results and high complication rates [11-13]. In spinal deformity surgery, nonfusion techniques with growing rods have been used for patients diagnosed with progressive early-onset scoliosis (EOS) or neuromuscular scoliosis (NMS) [14-16]. The rods function as an “internal brace” of the spine and aim to control or reduce spinal deformity. These minimally invasive nonfusion surgical techniques have been developed to limit the progression of the curve and to improve the thoracic volume while allowing the spine and thorax to grow along with the natural growth of the patient [17].

### Proposed Surgical Technique

The surgical treatment described in this protocol is the standard treatment protocol for scoliosis surgery, including perioperative antibiotic prophylaxis, a supine position on the spine table, and intraoperative motor-evoked potential (MEP) and somatosensory-evoked potential (SSEP) monitoring. Traction is applied to the patient’s head using a Mayfield skull clamp, and distal traction (10% to 15% of body weight) is applied with boots on the legs to achieve intraoperative correction. The level of instrumentation is determined according to the principles developed by Lenke [18,19].

**Figure 1:**
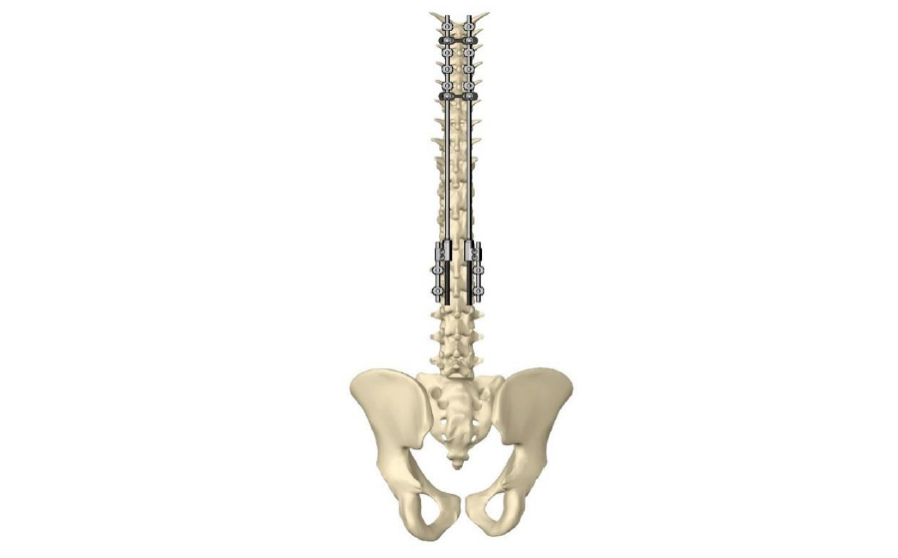
Example of NEMOST growing domino and E-SPINE system construct.

A total of 2 short 5-10–cm midline incisions are placed over the high thoracic and thoracolumbar anchor points. In the high thoracic region, 4 pedicle screws are placed. Additionally, 2 transverse process hooks are placed at the upper-level vertebra to prevent proximal junction kyphosis. At the thoracolumbar anchor region, 4 pedicle screws are placed, including a short rod on both sides (Figure 1).

**Figure 2:**
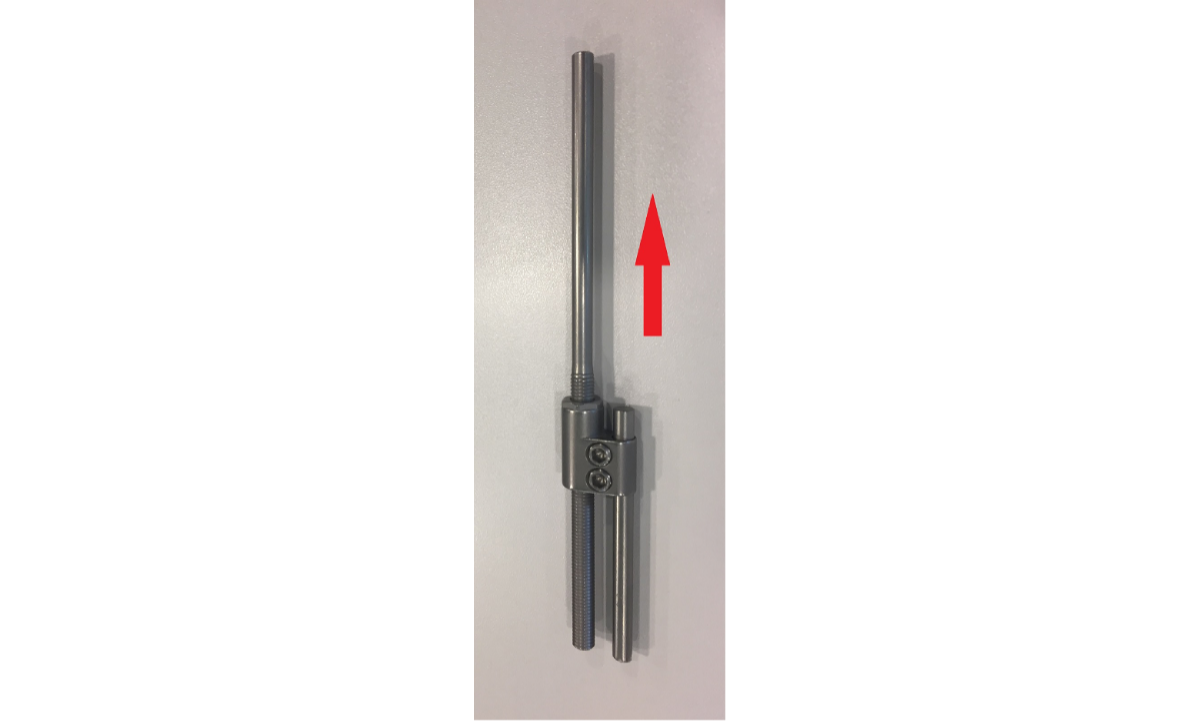
The one-way self-expanding rod (OWSER) and domino registered as NEMOST rod.

The one-way self-expanding rods (OWSERs) and domino is a CE-marked commercially available form of instrumentation registered as a NEMOST rod (E-SPINE, Euros; corresponding certificate: N°CE639319; Figure 2).

The OWSER is inserted into the subfascial plane between the 2 incisions from the caudal to the cranial region, guided by a cranio-caudal introduced chest tube under the superficial fascia. Subsequently, the OWSERs are fixed to the cranial anchors with an additional cross connector between the 2 rods. After the insertion of the rod, a moderate concave distraction is performed before the OWSER is connected to the laterally positioned small smooth thoracolumbar rods.

The initial correction of the scoliotic curve is obtained via intraoperative traction. The sagittal plane correction is obtained as a result of kyphotic rod contouring before rod insertion. The notched ratchet part of the rod, including the temporarily fixed domino connector that is positioned downward, remains straight to allow future elongation. The one-way self-expanding domino connector slides passively and gradually (1-mm step ratchet) along the notched part of the long rod. Rotation of the rod in the domino connector is prevented by a flat part of the notched rod (Figure 2). Postoperatively, patients have unrestricted mobilization during normal daily activities. Patients have follow-up appointments that adhere to the standard protocol of postoperative scoliosis care (Table 1).

**Table 1:** Scheme used during clinical care in children treated with nonfusion one-way self-expanding rods.

	Screening (1-2 months preoperative)	30 days preoperative	Preoperative	Postoperative clinic	2 months post surgery	Every 6 months postoperative till 2 years after skeletal maturity
Explanation providing informed consent	✓					
Sign informed consent		✓				
Examination treating MD		✓			✓	✓
X-rays	✓		✓	✓	✓	✓
Clinical picture spine (standing position)		✓				✓
SRS^a^ questionnaire		✓			✓	✓

^a^SRS: Scoliosis Research Society.

The minimally invasive nature of the procedure, combined with the system’s ability to allow the patient’s natural growth while working as an internal brace, holds promise for improvement in the treatment of progressive AIS. This aligns with earlier results that have been shown for patients with NMS and EOS [14,20].

To the best of our knowledge, a minimally invasive posterior spinal nonfusion (PSnF) surgery with OWSER has not previously been used in patients with AIS.

This study describes a study protocol for a clinical trial. This study aims to evaluate the efficacy and safety of a nonfusion bipolar self-expanding rod system, here referred to as the “nonfusion OWSER system,” in the surgical treatment of patients with progressive AIS. Based on the positive surgical results and relatively low complication rates found when this type of system is used in patients with NMS or EOS [14,16,20], we hypothesize that the minimally invasive surgery using the nonfusion one-way self-expanding rod system (nonfusion OWSER) system is safe and efficient for the surgical correction of progressive AIS.

## Methods

### Ethical Considerations

The study protocol (version 3, October 29, 2021) describes a single-center cohort study. Ethics approval was granted by the Medical Ethical Committee of the Amsterdam University Medical Center (UMC) in the Netherlands, on May 20, 2020 (METC 2020_023, NL67124.018.19). Due to the COVID-19 pandemic, we were unsure if the study would be able to continue. This has led to a delay in the publication of this study protocol. This study will be conducted according to the principles of the Declaration of Helsinki (2013) and in accordance with the Medical Research Involving Human Subjects Act (WMO). It is registered in the Dutch trial register (NL8659) and the international trial register (NCT04441411). On October 14, 2023, a total of 5 patients have been included in the prospective study.

### Participants

All patients will be recruited by 1 single surgeon embedded in a multidisciplinary scoliosis team in the Amsterdam UMC in the Netherlands. Both patients and patients’ legal guardians must sign an informed consent form before the patient is included in the study. The inclusion criteria are (1) a diagnosis of AIS, (2) an age of >7 years, (3) a primary Cobb of >30°, (4) failed nonoperative treatment, (5) a Risser grade of ≤2 and a skeletal age of hand and wrist x-rays of ≤14 years, (6) a nonrigid curve, (7) all Lenke types, and (8) no previous spinal surgery.

A patient who meets any of the following criteria will be excluded from participation in this study: (1) nonidiopathic scoliosis: musculoskeletal or neurologic conditions responsible for the cause of the spine curvature (ie, neuromuscular, congenital, or syndromic scoliosis); (2) history of previous spine surgery; (3) Risser grade >2; (3) disease or deformity likely to affect the stability of the device (ie, inadequate anatomy of pedicles, trauma or tumor in fixation region, severe osteoporosis, bone destruction, or poor bone quality); (4) nonreducible scoliosis; (5) known allergy or intolerance to one of the device material; (6) acute or chronic infections, local or systemic; (7) absence or insufficiency of covering tissues; or (8) pathological obesity.

### Data Collection and Statistical Analyses

Data will be collected at baseline, directly post surgery, after 2 months, and every 6 months after the operation until 2 years after skeletal maturity (see Table 1).

To describe the included patient group, patient characteristics such as age at surgery, sex, height, weight, medical and surgical history, blood loss during surgery, operative time, length of hospitalization, upper and lower level of fixation, short- and long-term complications (surgical site infections, neurological complications, implant failure, proximal junctional kyphosis, etc), and when applicable, secondary surgery (including the time between the first and second surgery) will be extracted from the patient's medical record. The data will be summarized in tables listing the mean, SD, median, minimum, maximum, and number of subjects for continuous data or in tables listing the quantities and percentages for categorical data, where appropriate.

For the primary goal, to analyze the efficacy of OWSER, the correction of the scoliotic curve will be measured by comparing the preoperative and postoperative Cobb angle with the Cobb angle 2 years after maturity. Radiological parameters will be measured by an orthopedic resident, trained to measure all radiological parameters. Measurements will be randomly verified by a senior experienced spine surgeon. Measured radiological parameters include the main Cobb angle (measured as defined by Cobb [21]), compensatory Cobb angle, correction after surgery, sagittal balance (thoracic kyphosis, lumbar lordosis, and proximal and distal junctional angle), pelvic parameters (pelvic obliquity, sacral slope, pelvic incidence, and pelvic tilt), Risser sign, and vertebral rotation based on full spine standing posterior-anterior and lateral radiographs. Differences between the Cobb angles and growth will be analyzed using repeated ANOVA measures. To correct for possible confounders, a mixed-model regression analysis will be performed including age at surgery, Risser sign, weight, and sex.

Patient satisfaction and function will be assessed using patient appearance (shoulder imbalance and pelvic imbalance) and patient-reported outcome measurements using the Scoliosis Research Society's-22r (SRS-22r) questionnaire. This questionnaire is provided in Dutch and the use of the questionnaire has previously been validated [22]. The safety of the surgical device will be monitored and registered based on (serious) adverse events and complication rates. Radiological and patient-related outcomes will be used to assess the clinical outcomes and with this, the efficiency of the implant. These parameters will be measured at every follow-up moment (Table 1).

In case of missing values at the follow-up, the patient will remain included in the study. Based on the amount of missing values, the patients will be excluded from the calculations from the specific follow-up moment. This will be described in the results section of the specific study. A *P* value of <.05 will be regarded as statistically significant.

### Adverse Events

Adverse events (AEs) and serious adverse events (SAEs) are documented according to the definition of the Dutch Ethics Committee and will be acted on accordingly. AEs are defined as any undesirable experience occurring to a subject during the study, whether or not considered related to the investigational product. These will be actively reported by the subject or observed by the investigator or his staff. All AEs will be recorded throughout the study, and their severity and relation to the investigational device will be determined.

SAEs are documented as any untoward medical occurrences or effects that result in death, are life-threatening (at the time of the event), require hospitalization or the prolongation of existing inpatients’ hospitalization, in persistent or significant disability or incapacity, are congenital anomalies or birth defects, or involve any other important medical events that did not result in any of the outcomes listed above due to medical or surgical interventions but that could have been based upon appropriate judgment by the investigator.

The research and treatment team must report any SAEs via telephone or email correspondence within 24 hours after the occurrence of the event to the principal investigator and the delegated medical board of directors. A completed initial SAE report must be sent, as indicated in the study’s case report form.

### Data Management

All data will be handled confidentially according to the General Data Protection Regulation (GDPR) guidelines. Research subjects will receive a 3-digit ID number upon beginning the study. One patient identification log (Castor electronic data capture will be maintained separately to identify the research subjects and will only be accessible to the principal investigator and the project leader.

### Sample Size

To perform a sample size analysis, we compared our study to the study conducted by Miladi et al [23]. The sample size calculation is based on an ANOVA analysis and calculated using the statistical program G*Power (Heinrich-Heine-Universität Düsseldorf) [24,25].

Miladi et al [23] reported the results from the precursor of our OWSER system from the higher thoracic spine to the pelvis in 100 patients with NMS. The mean Cobb angle improved from 89° (range, 25°-149°) to 35° (range, 6°-53°) 3 years after surgery [23]. No SDs were reported. In light of this study, we expect a Cobb angle of 35° at maturity. To use these numbers for the sample size calculation, we assumed a normal distribution of the data; as such, the SDs of the preoperative Cobb angle were 31 and 11.75 for the postoperative Cobb angle [26]. We assumed a correlation of 0.5 and an effect size of 0.4 based on the correction described by Miladi et al [23]. The sample size calculation indicated that 12 patients ought to be included to examine the significant difference between the preoperative Cobb angle and the Cobb angle at the latest follow-up (power: 80%, significance level: 0.05).

Additionally, 20% more patients were included to account for a possible loss of subjects in the follow-up. In conclusion, the highest sample size indicated was 12; therefore, the minimal sample size needed for this study to address the primary goal is 14 patients. After the inclusion is completed, a post hoc power analysis will be performed to conclude if a significant number of patients is included in the study.

## Results

Patient inclusion started on November 17, 2021, and we hope to finalize patient inclusion by the beginning of 2025. The first results will be expected by the beginning of 2024. On October 14, 2023, a total of 5 patients have undergone surgical correction with the proposed surgical technique. We aim to publish the 2-year results of these patients in May 2024.

## Discussion

### Principal Findings

Currently, all patients with AIS and diagnosed with curves exceeding 40° to 45° are recommended surgical correction followed by PSF to fix the patients’ spine for the rest of their lives [2,3]. In this study protocol, we describe a protocol to investigate the safety and efficacy of a minimally invasive posterior bipolar nonfusion OWSER for the surgical treatment of patients diagnosed with AIS.

The concept of PSnF for scoliosis surgery is not new. For patients diagnosed with EOS and NMS, multiple successful PSnF options are available and are currently regarded as the standard surgical treatment [16]. However, to the best of our knowledge, this concept has not been used in patients diagnosed with AIS.

The aim of this prospective study is to determine the efficiency and safety of the implant by analyzing radiological parameters (primarily Cobb angle), patient satisfaction scores, and complication rates. The expected primary benefit of this surgical technique is an efficient surgical correction of the Cobb angle without PSF. Because of the minimally invasive character of the surgical procedure, the spinal structures of the apex of the curvature between the thoracic and lumbar anchor points are not altered. Although some cases of auto-fusion have been described when using minimally invasive surgery [14], to the best of our knowledge, no complete full spinal fusion will occur. This might only allow the removal of the nonfusion OWSER at the end of spinal growth in patients in which the curve remains stable for a longer period of time and remains under 30° [27]. In addition, the system allows for a natural correction of the spinal deformity while the correct balance and alignment of the spine are maintained. Because of its self-expanding function, the system allows the patient to follow its final natural growth while functioning as an “internal brace.” Finally, due to the minimally invasive nature of the surgical procedure, the overall surgical time and general anesthesia time will be reduced, and the scar length can be limited to two scars that are 5-10 cm in length at the thoracic and lumbar fixation level.

For patients diagnosed with NMS, the positive long-term results of a nonfusion OWSER were described by Gaume et al [20] in 2021. The original nonfusion OWSER provides instrumentation for NMS from T1 to the sacrum. In patients with AIS, however, instrumentation is only needed for the length of the structural curve. Therefore, we suggest inserting the notched rod at the end of the caudal part of the construct. Hereby, the thoracolumbar approach is performed using a smaller incision. The OWSER instrumentation elongates and corrects the deformity spontaneously alongside the natural growth of the patient, allowing spinal balance correction; it does not touch the apex vertebrae of the spinal curvature and prevents complete PSF. The OWSER instrumentation can be considered an “internal brace.”

Although positive long-term results have been published following PSnF surgery in patients with NMS, complications have been recognized. Gaume et al presented their preliminary results of the use of a minimal invasive OWSER for the treatment of patients with NMS. They found an overall complication rate of 38% (n=8), consisting of 14% (n=3) mechanical complications and 24% (n=5) infectious complications [20]. Although these complication rates seem high, compared to the traditional growing techniques, there is an important decrease in complication rates [28].

All of the included patients were fixed from the high thoracic level up to the pelvis. Our patient population will arguably be fixed over a shorter number of vertebrae, lowering the amount of force distributed over the system. In addition, Gaume et al [20] found a postoperative infection rate of 24% (n=5) directly post surgery. In postoperative patients diagnosed with AIS, this would be unacceptable. However, these results cannot be directly translated to our patient population, as it is known that patients with NMS have poor general status [29]. This makes them more prone to infectious complications as compared to the healthy patients with AIS who will be included in this study.

Other possible complications are an inherent part of spinal surgery, such as intraoperative cardiologic complications and neurological complications. The risk of neurological complications will be minimized using intraoperative neuromonitoring via both MEP and SSEP during the surgical procedure. All AE and SAE are monitored based on the guidelines of the Dutch Ethics Committee, and will help determine the safety of the proposed surgical technique.

The absence of a control group could be seen as a potential limitation. In this study, we do not have a control group as we focus on the efficiency and safety of the surgical device. If the device is proven to be efficient and safe, a follow-up will be proposed to compare the postoperative results between the nonfusion OWSER system and the traditional PSF. In addition, this would allow us to discuss the cost-effectiveness of the PSnF surgery compared to the traditional PSF surgery.

### Conclusions

In conclusion, this study is designed to evaluate the efficiency and safety of the surgical treatment with a bilateral bipolar OWSER implant in patients with progressive AIS. We hypothesize that the system is safe and efficient in the surgical treatment of AIS, while it serves as an “internal brace,” allowing the patients to follow their natural growth and individual balance. To the best of our knowledge, this is the first study to investigate the potential efficacy and benefits of the use of the nonfusion OWSER in patients diagnosed with AIS who failed conservative treatment.

## Abbreviations

AE: adverse event
AIS: adolescent idiopathic scoliosis
EOS: early-onset scoliosis
GDPR: General Data Protection Regulation
MEP: motor-evoked potential
NMS: neuromuscular scoliosis
nonfusion OWSER: nonfusion one-way self-expanding rod system
OWSER: one-way self-expanding rods
PSF: posterior spinal fusion
PSnF: posterior spinal nonfusion
SAE: serious adverse event
SRS-22r: Scoliosis Research Society's-22r
SSEP: somatosensory-evoked potential
UMC: University Medical Center

## Data Availability

The data sets generated and analyzed during this study are not publicly available due to the fact that the study has not been finalized and data collection has not been completed yet, but are available from the corresponding author on reasonable request.
